# Doxycycline Attenuates Cancer Cell Growth by Suppressing NLRP3-Mediated Inflammation

**DOI:** 10.3390/ph14090852

**Published:** 2021-08-26

**Authors:** Mohammad Alsaadi, Gulcin Tezcan, Ekaterina E. Garanina, Shaimaa Hamza, Alan McIntyre, Albert A. Rizvanov, Svetlana F. Khaiboullina

**Affiliations:** 1 Institute of Fundamental Medicine and Biology, Kazan Federal University, 420008 Kazan, Russia; mhmad.alsadi19955@hotmail.com (M.A.); kathryn.cherenkova@gmail.com (E.E.G.); shaimaa.hamza@mail.ru (S.H.); rizvanov@gmail.com (A.A.R.); 2 Department of Fundamental Sciences, Faculty of Dentistry, Bursa Uludag University, Bursa 16059, Turkey; 3 Centre for Cancer Sciences, Faculty of Medicine and Health Sciences, University of Nottingham, Nottingham NG7 2RD, UK; Alan.Mcintyre@nottingham.ac.uk; 4 Department of Microbiology and Immunology, University of Nevada, Reno, NV 89557, USA

**Keywords:** doxycycline, nod-like receptor protein 3 (NLRP3), inflammasome, apoptosis, cancer

## Abstract

NLR family pyrin domain containing 3 (NLRP3) inflammasome formation is triggered by the damaged mitochondria releasing reactive oxygen species. Doxycycline was shown to regulate inflammation; however, its effect on NLRP3 in cancer remains largely unknown. Therefore, we sought to determine the effect of doxycycline on NLRP3 regulation in cancer using an in vitro model. NLRP3 was activated in a prostate cancer cell line (PC3) and a lung cancer cell line (A549) before treatment with doxycycline. Inflammasome activation was assessed by analyzing RNA expression of NLRP3, Pro-CASP-1, and Pro-IL1β using RT-qPCR. Additionally, NLPR3 protein expression and IL-1β secretion were analyzed using Western blot and ELISA, respectively. Tumor cell viability was determined using Annexin V staining and a cell proliferation assay. Cytokine secretion was analyzed using a 41Plex assay for human cytokines. Data were analyzed using one-way ANOVA model with Tukey’s post hoc tests. Doxycycline treatment decreased NLRP3 formation in PC3 and A549 cells compared to untreated and LPS only treated cells (*p* < 0.05). Doxycycline also decreased proliferation and caused cell death through apoptosis, a response that differed to the LPS-Nigericin mediated pyroptosis. Our findings suggest that doxycycline inhibits LPS priming of NLRP3 and reduces tumor progression through early apoptosis in cancer.

## 1. Introduction

Inflammation is a cancer risk factor [1]. Recent studies have demonstrated that inflammation involves formation of a functional inflammasome that supports the release of inflammatory cytokines [2]. One of these is the Nod-like receptor protein 3 (NLRP3) inflammasome, which facilitates inflammation through the release of pro-inflammatory cytokines, IL-1β or IL-18 [3]. It is proposed that the NLRP3 inflammasome contributes to chronic inflammation, malignant transformation and carcinogenesis in patients and animal models [4,5,6,7,8,9,10]. Therefore, targeting the NLRP3 inflammasome could be an approach to overcome pro-tumorigenic inflammation and provide an option for the development of novel therapeutics for cancer treatment [11].

Dysfunction of mitochondria in tumor cells could be a source of chronic inflammation and inflammasome activation [12]. Supporting this notion, generation of reactive oxygen species by the mitochondria was shown to trigger NLRP3 inflammasome formation in sepsis models and inflammatory diseases associated with mitochondrial damaged [13,14]. These data suggest that therapeutics targeting mitochondrial activity could interfere with this organelle contribution to chronic inflammation. In line with this, antibiotics targeting mitochondrial activity present an attractive tool to regulate inflammasomes. This is based on the similarity between bacteria and mitochondria, where antibiotics could target this eukaryotic organelle and modulate NLRP3 activity [15]. There are some antibiotics that affect inflammasome activity. For example, nigericin, a polyether antibiotic that targets ion transport and ATPase activity in mitochondria, and activates NLRP3 [16]. Additionally, other antibiotics such as polymyxin B, gramicidin, tyrothricin, and neomycin have been demonstrated to activate the inflammasome [17].

Antibiotics have been assessed previously in cancer therapy because of their anti-proliferative, pro-apoptotic, and anti-epithelial-mesenchymal-transition (EMT) potential [18]. Recent studies identified that antibiotic use in cancer therapy can also change the microbiome, reduce the body’s immune defense and promote inflammation [19]. Our previous study demonstrated that nigericin induced NLRP3 inflammasome-mediated pyroptosis [10]. However, the cell death varied depending on NLRP3 activation level and cytokine release. While nigericin induced cell death in tumor cells producing low levels of IL-1β and IL-18, increased proliferation was found in cancer cells producing, high levels of IL-1β and IL-18 [10]. Our findings prompt further investigation of the impact of antibiotic modulation of NLRP3 activity to determine the long-term effects of this. 

Doxycycline is a broad-spectrum tetracycline-class antibiotic used to treat cancer-associated infections [20,21,22,23,24]. Doxycycline interferes with bacterial translation by targeting the 30S ribosome of bacteria and inhibiting protein production. The result is either bacteriostatic or bactericidal [20]. Doxycycline was shown to have a therapeutic effect against leptospirosis-induced NLRP3 inflammasome priming via upregulation of the Na/K-ATPase Pump β1 subunit [25]. However, the effect of doxycycline on NLRP3 in cancer cells remains unknown. Therefore, in this study we selected a prostate cancer cell line, PC3, and lung cancer-derived cell line, A549, to investigate the impact of doxycycline on NLRP3 activation. Selection of these cell lines was based on our previous data demonstrating opposite effect on inflammasome activation [10]. PC3 and A549 cells were treated with doxycycline to investigate the role of doxycycline on NLRP3 activity and cell proliferation based on IL-1β release. 

## 2. Results

**Figure 1:**
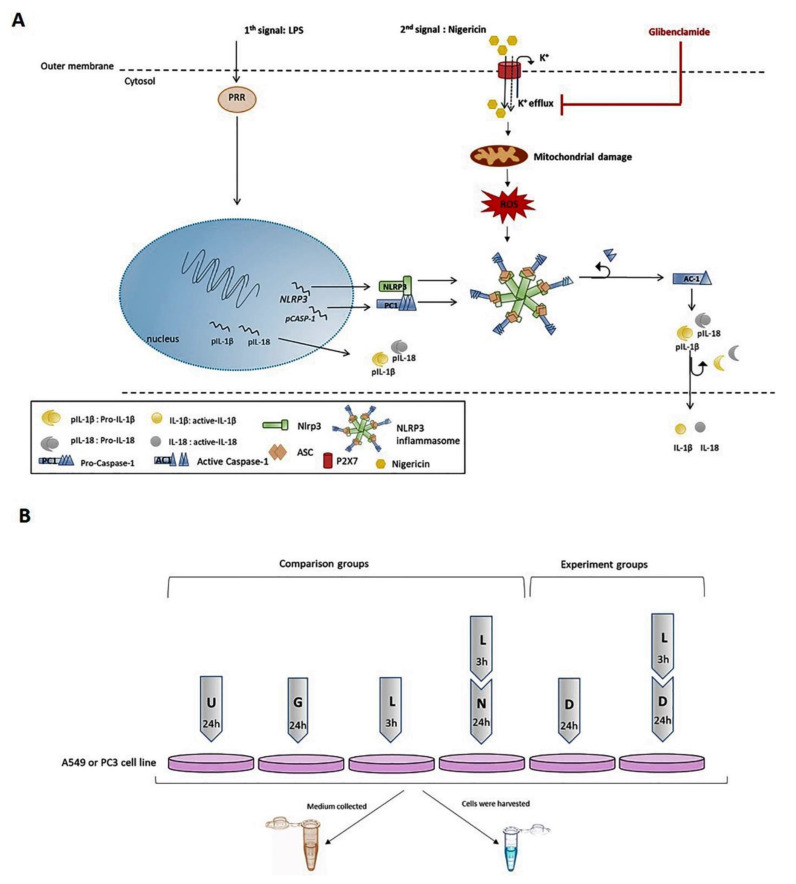
The experimental design. (**A**) The effect of LPS, Nigericin and Glibenclamide on NLRP3 activation. LPS as the signal 1 is a priming trigger inducing the NLRP3 and pro-IL-1β production. Nigericin is the signal 2 and plays role in polymerization of an active NLRP3 inflammasome complex which recruits pro-Caspase-1 and cleaved it to active form of Caspase-1. The Caspase-1 liberates functional IL-1β and IL-18, regulatory cytokines of inflammation. Glibenclamide is a potassium channel inhibitor and blocks the polymerization of NLRP3 protein complex. (**B**) The experimental control groups consisted of: 1. A549 and PC3 cells treated with glibenclamide for 24 h as the positive control of NLRP3 inhibition; 2. LPS-only for 3 h as the positive control of NLRP3 priming; 3. LPS+nigericin treatment for 24 h as the positive control of active NLRP3 inflammasome complex. The experimental test groups consisted of A549 and PC3 cells treated with doxycycline-only for 24 h and pretreated with LPS for 3 h. U: Untreated PC3 or A549 cells, G: Glibenclamide, L: LPS, N: Nigericin, D: Doxycycline, PRR: The cytosolic pattern recognition receptor, AC1: Active Caspase-1, PC1: Pro-Caspase-1, ASC: Apoptosis-associated speck-like protein containing a CARD, ROS: reactive oxygen species.

NLRP3 activation was induced by two stimuli: priming and activating [9,26] as described in Figure 1. LPS (1 µg/mL; Sigma, St. Louis, MO, USA) treatment for 3 h was used as priming stimulus. Nigericin (20 µM; Sigma, St. Louis, MO, USA) was added for 24 h as the second stimulus [10]. Glibenclamide (25 µg/µL; Invivogen, San Diego, CA, USA) was used to inhibit the inflammasome [27] (Figure 1A). A description of the experimental groups used in this study can be found in Figure 1B. Doxycycline (20 µg/mL; Sigma, St. Louis, MO, USA) was added after LPS treatment to analyze itse effect on NLRP3 activation (Figure 1B). 

### 2.1. Doxycycline Attenuates NLRP3 Inflammasome Activation

**Figure 2:**
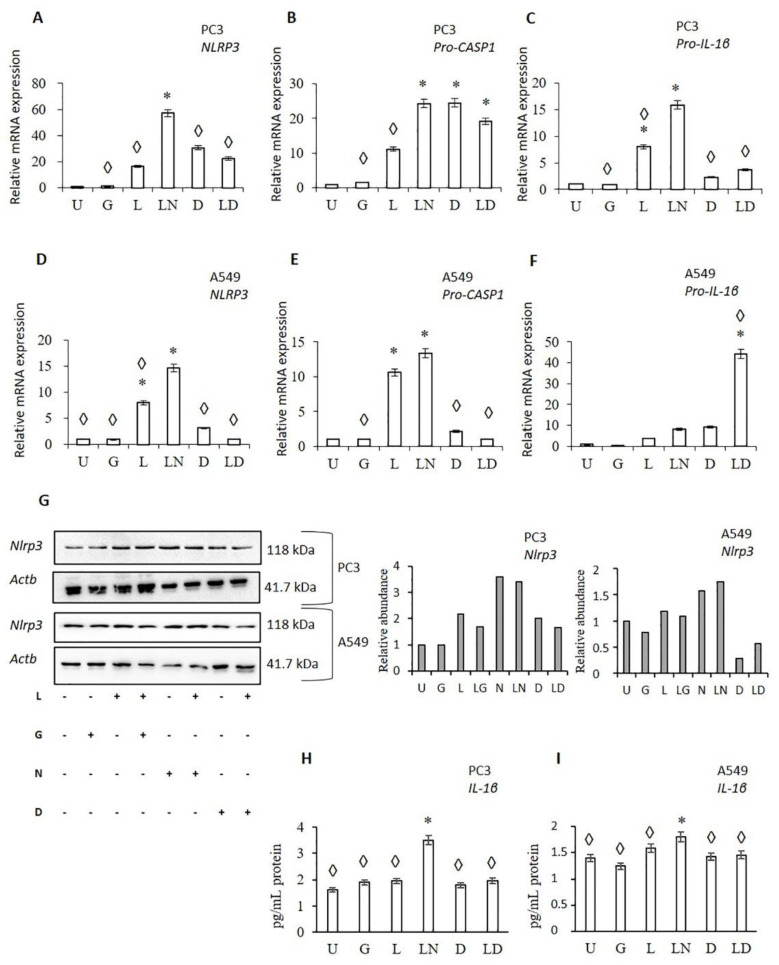
The effect of doxycycline on NLRP3 inflammasome activation. Doxycycline effects on: (**A**–**C**) The RNA levels of NLRP3 downstream genes in PC3 (**A**–**C**), and A549 cells (**D**–**F**). The effect of doxycycline on NLRP3 protein translation (**G**), cell-secretion of IL-1β in PC3 (**H**), and A549 cells (**I**). U: Untreated, G: Glibenclamide, L: LPS, LN: LPS-Nigericin, D: Doxycycline, LD: LPS Doxycycline. * adjusted *p* < 0.05 compared to untreated cells; ◊: adjusted *p* < 0.05 compared to LN treated cells. Data were analyzed using one-way ANOVA and Tukey’s post hoc tests (*n* = 3).

To analyze the effect of doxycycline on NLRP3 activation, the RNA expression of NLRP3, pro-CASP1, and pro-IL-1β was investigated in PC3 and A549 cells treated with doxycycline, LPS and combination LPS-doxycycline (Figure 2). As anticipated, LPS only and LPS-nigericin significantly increased NLRP3 and pro-CASP1 expression in PC3 and A549 cells compared to untreated cells. Larger differences in NLRP3 and Pro-CASP1 expression was found in PC3 compared to A549 cells (Figure 2A–E). LPS-nigericin treatment increased NLRP3 RNA expression in PC3 and A549 cells 57.15 and 14.58 fold, respectively (Figure 2A,D). LPS-Nigericin treatment also increased levels of Pro-CASP1 in PC3 and A549 cells 24.24 and 13.36 fold, respectively, compared to untreated controls (Figure 2C,F). These findings suggest that treatment with LPS only or combined LPS-Nigericin activates the NLRP3 inflammasome in both PC3 and A549 cells, although to a different degree in each cell line (Figure 2A–F). An increased expression of NLRP3 protein was also found in cells treated with LPS only and LPS-Nigericin compared to untreated PC3 and A549 cells (Figure 2G). In contrast, glibenclamide, an inhibitor of NLRP3, did not affect the RNA expression of NLRP3, pro-CASP1, and pro-IL-1β as compared to untreated PC3 and A549 cells (Figure 2A–F). 

In both, PC3 and A549 cells, doxycycline increased NLRP3 and pro-CASP1 RNA expression compared to untreated controls (Table S1). However, when compared to LPS, the doxycycline induction of NLRP3 and pro-CASP1 RNA were significantly lower (*p* < 0.05) in A549 cells (Figure 2D–E). Additionally, LPS-doxycycline treatment reduced NLRP3 and pro-CASP1 expression compared to LPS only and LPS-nigericin treated A549 cells (*p* < 0.001) (Figure 2D–E). Doxycycline treatment showed a similar effect on the expression of NLRP3 and Pro-CASP1 in PC3 cells, but changes were not significant compared to controls (Table S1). 

Doxycycline only and LPS-doxycycline treatment induced pro-IL-1β RNA expression compared to untreated PC3 and A549 cells. However, this induction was significantly lower than that in LPS only and LPS-nigericin treated PC3 cells (Figure 2C). Additionally, LPS-doxycycline combined treatment significantly increased pro-IL-1β RNA expression in A549 cells compared to LPS only, doxycycline-only, and LPS-nigericin treated cells (Figure 2C,F). As expected, doxycycline only and LPS-doxycycline treatment decreased secretion of IL-1β in both, PC3 and A549 cells compared to LPS only and LPS-Nigericin treated cells (Figure 2H,I). Collectively, these findings suggest that doxycycline has an inducing effect on pro-IL-1β transcription. However, it reduces NLRP3 inflammasome protein synthesis leading to decreased pro-IL-1β maturation. 

### 2.2. Doxycycline Decreased Cell Proliferation and Viability

**Figure 3:**
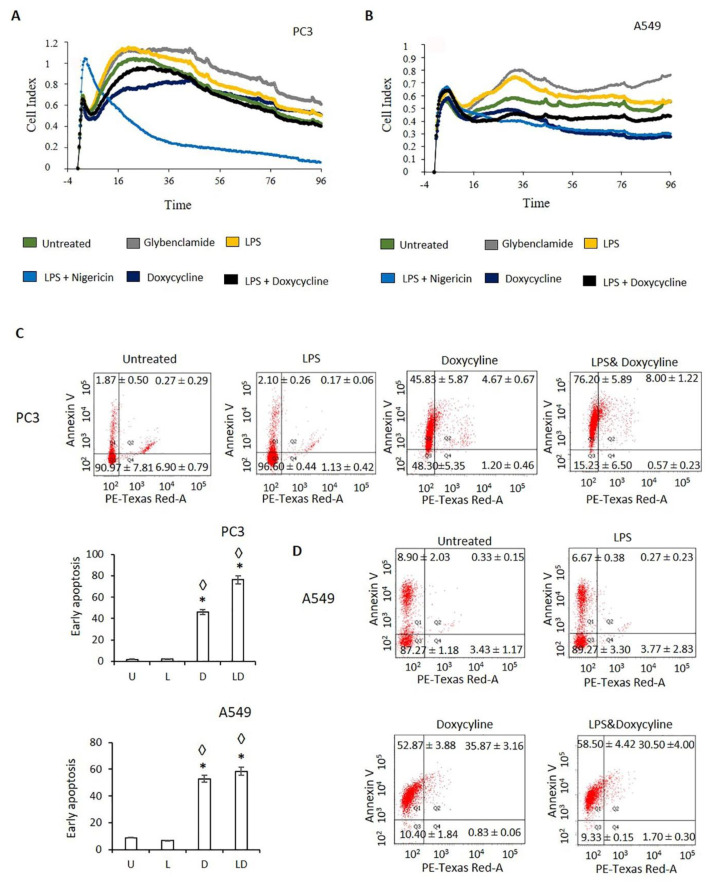
The effect of doxycycline mediated inhibition of NLRP3 on proliferation and viability of cancer cells. (**A**) Doxycycline and LPS-doxycycline effect on PC3 cell proliferation; (**B**) Doxycycline and LPS-doxycycline effect on A549 cell proliferation; (**C**) PC3 cell viability, (**D**) A549 cell viability. The adjusted p-values were calculated using one-way ANOVA and Tukey’s post hoc tests. U: Untreated, L: LPS, D: Doxycycline, LD: LPS-Doxycycline. * *p* < 0.05 compared to untreated cells ◊ *p* < 0.05 compared to LPS treated cells; *n* = 3.

PC3 and A549 cells were treated with LPS, doxycycline, LPS-doxycycline, glibenclamide, and LPS-nigericin combinations to analyze the impact on cell proliferation in real-time using the xCELLigence biosensor array for 96 h. Additionally, PC3 and A549 cells as well as HEK293T cells, as non-tumor epithelial cells, were treated with LPS, doxycycline and LPS-doxycycline combinations, as described in Figure 1B and Figure S1, to determine the impact on cell viability and the nature of the cell death induced (pyroptosis or apoptosis) by Annexin V assay. PC3 and A549 cell viability kinetics indicated that glibenclamide slightly increased cell proliferation rate compared to untreated cells. NLRP3 activation with LPS did not affect PC3 and A549 cell proliferation. In contrast, LPS-nigericin substantially attenuated cell proliferation of PC3 and A549 cells (Figure 3A,B). These findings suggest that increased NLRP3 expression by Nigericin coincides with an arrest of cell proliferation. 

Doxycycline only and LPS-doxycycline treatment reduced PC3 and A549 cell proliferation compared to untreated, glibenclamide only and LPS only treated cells (Figure 3A,B). Additionally, doxycycline and LPS-doxycycline treatment reduced the viability of PC3 and A549 cells and increased early apoptosis (Annexin V (+)/PE (−)) compared to untreated and LPS treated cells (Figure 3C,D and Table S2). It was noted that the proliferation of doxycycline only and LPS-doxycycline treated cells was higher than those treated with LPS-nigericin (Figure 3A,B). Doxycycline only and LPS-doxycycline treatments did not affect the viability of HEK293T cells (Figure S1). 

### 2.3. The Effect of Doxycycline on Cytokine Secretion

**Figure 4:**
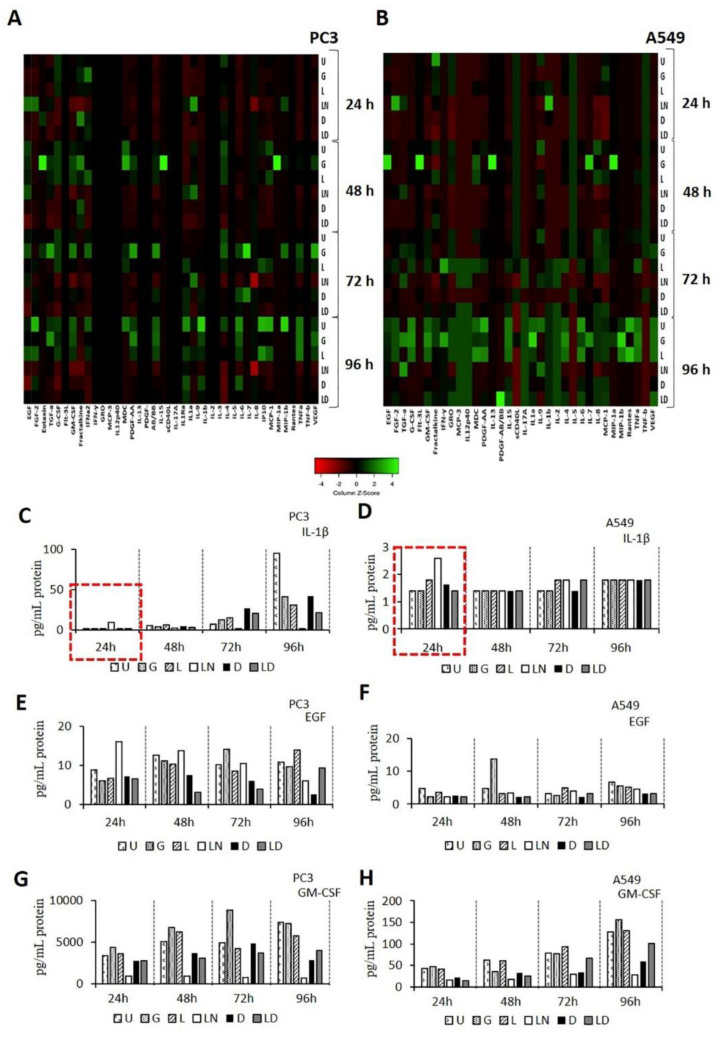
Effect of Doxycycline on cytokine release pattern in PC3 and A549 cells. (**A**,**B**). The heat map graphs of cytokine release pattern in PC3 and A549 cells. (**B**,**C**). The time-dependent secretion of IL-1β. (**D**–**H**). The time-dependent secretion of EGF and GM-CSF in PC3 and A549 cells. U: Untreated, G: Glibenclamide, L: LPS, LN: LPS-Nigericin, D: Doxycycline, LD: LPS-Doxycycline.

Cell culture medium was collected 24–96 h after treatment with glibenclamide, LPS, LPS-nigericin, doxycycline and LPS-doxycycline combinations, and used to assess the expression of 41 cytokines in each of these conditions. Cytokine levels for each of these conditions in PC3 and A549 are summarized in Figure 4A,B, Table S3 and Table S4. Levels of cytokines in glybenclamide treated cells remained similar to controls. LPS-nigericin treatment increased secretion of IL-1β at 24 h, while after this, the levels of IL-1β decreased in both cell lines. Doxycycline and LPS-doxycycline treatment decreased the levels of IL-1β for the first 24 h. (Figure 4C,D). Additionally, doxycycline and LPS-doxycycline treatments decreased EGF, FGF2, TGFA, and GM-GSF levels compared to LPS and LPS-Nigericin treated PC3 and A549 cells at the same time point. The inhibitory effect of doxycycline and LPS-doxycycline treatment on EGF and GM-CSF was constant for 96 h compared to LPS-only-treated cells (Figure 4E–H).

## 3. Discussion

Doxycycline is one of the tools used for cancer management. It is used to reduce the risk of cancer-associated infections [23,24,28,29]. Additionally, doxycycline is employed for differential diagnosis between tumors and infections with similar symptoms. For instance, prostatic inflammation causes increased serum PSA levels similar to that in prostate cancer [30,31,32,33]. In this example, the reduction in PSA levels to normal after doxycycline treatment indicates prostatitis, not prostate cancer [34]. Additionally, during the last two decades, many studies have highlighted the cell death-inducing effect of doxycycline in a range of tumor cells, including breast prostate, pancreas, lung, and colorectal cancers and leukemia [35,36,37,38,39]. A recent study linked the anti-cancer activity of doxycycline to its effect of inhibiting mitochondrial protein synthesis and the inner mitochondrial membrane potential (ΔΨm) [40]. Decreased ΔΨm was shown to lower apoptotic threshold [39] and induce cell death through pyroptosis [41]. 

Pyroptosis is a programmed inflammatory cell death induced by inflammatory caspases [42]. A mature inflammatory caspase, such as pro-Caspase-1, is released by activated NLRP3 and subsequently cleaves pro-inflammatory cytokines, IL-1β and IL-18 [9,43]. Today it is evident, that many antibiotics commonly used for treatment of infectious diseases, can induce or reduce NLRP3 activity by targeting mitochondrial processes [15]. In addition, a recent study reported that the mechanism of doxycycline efficacy against Porphyromonas gingivalis infection could be explained by its inhibitory effect on NLRP3 and IL-1β production [40]. 

IL-1β released from tumors is strongly implicated in tumor progression [44,45]. While present at a low level, IL-1β can induce effective anti-tumor immunity and suppress tumor formation [44,46,47,48]. However, at a high-level IL-1β released by tumor cells promotes tumor progression and metastasis [44,46,47,48]. The effect of long-term use of antibiotics on IL-1β release by tumor cells is largely unknown. 

Previously, we demonstrated that tumor cells release various amounts of IL-1β based on their ability to activate NLRP3 [10]. According to our findings, PC3, a prostate cancer cell line, released the highest amount of IL-1β, while A549, a lung adenocarcinoma cell line, secreted low level of IL-1β [10]. Therefore, in this study, we selected these cell lines to investigate the effect of doxycycline on NLRP3 inflammasome activation and cell viability. Our results suggest that doxycycline has an inhibiting effect on LPS priming of the inflammasome, and that it can attenuate the production of pro-CASP1 and NLRP3 RNA and NLRP3 protein assembly irrespective of the level of IL-1β production and secretion in these tumor cells.

Nigericin is an antibiotic that acts as a K+ ionophore [49]. Nigericin triggered release of IL-1β has been demonstrated to be NLRP3-dependent [16]. Our previous study has shown that while nigericin induced tumor cell death, inhibitors of NLRP3, including VX765 and glibenclamide [50], promote tumor cell proliferation [10]. In this study, doxycycline-treated tumor cells tended to react similarly to those treated with nigericin, including exhibiting decreased cell proliferation compared to glibenclamide-treated cells. These changes in tumor cell proliferation could be linked to an increased cell death via apoptosis and pyroptosis [51]. In NLRP3 mediated pyroptotic cell death, both Annexin V and cell impermeant dyes can enter the cell from the opened membrane pores and localize in the inner membrane [51]. Nigericin-only and LPS-nigericin caused a lytic cell death, possibly pyroptosis, which is positive for both Annexin V and PI staining [10]. In contrast to the effect of Nigericin, we identified that doxycycline-only and LPS-doxycycline treatment induced cell death was positive for Annexin V but negative for PI uptake in cancer cells. These findings suggest that, although doxycycline reduces the ΔΨm of tumor cells [39], it decreases cell proliferation and induces apoptosis rather than pyroptosis. A study by Alexander-Savino and colleagues demonstrated that doxycycline induces cell death through the activation of caspase-8, suggesting doxycycline may induce apoptosis via a mitochondrial-independent pathway [52]. Additionally, apart from LPS-only and LPS-nigericin, doxycycline and LPS-doxycycline decreased the level of anti-apoptotic cytokines, such as EGF, FGF2, TGFA, and GM-GSF [53,54,55,56]. These results further support the ability of Doxycycline to inhibit LPS priming of NLRP3 activation, and indicate the inhibitory effect of Doxycycline on LPS-induced anti-apoptotic cytokine secretion in cancer. Additionally, studies showed that tumor cells are more affected by doxycycline than fibroblasts because of the faster depletion of the oxidative phosphorylation enzymes that maintain ΔΨm in tumor cells than non-tumor cells result [39]. Similarly, our findings suggest that doxycycline-only and LPS-doxycycline treatment did not affect non-tumor epithelial cells, making doxycycline a well-tolerated drug for cancer therapy. 

## 4. Materials and Methods

### 4.1. Cell Lines and Maintenance

PC3, a grade IV adenocarcinoma prostate cell line, A549, a non-small cell lung cancer (NSCLC) cell line and HEK293T, a human embryonic kidney cell line as a non-tumor epithelial cell line, were available from the American Type Culture Collection (ATCC; Rockville, MD, USA). Cells were grown in the standard cell culture medium, Dulbecco’s Modified Eagle’s Medium-F12 (PanEco, Moscow, Russia), supplemented with 10% fetal bovine serum (FBS) (HYCLONE, Logan, UT, USA), 50 U/mL of penicillin and 50 µg/mL of streptomycin (PanEco, Moscow, Russia), 2 mM L-glutamine and 1 mM sodium pyruvate (PanEco, Moscow, Russia). Cells were incubated in a 5% CO_2_ humidified incubator at 37 °C.

### 4.2. Reagents

Lipopolysaccharides (LPS) from Escherichia coli O111:B4, nigericin and doxycycline were purchased from Sigma (St. Louis, MO, USA). Glibenclamide was available from Invivogen (San Diego, CA, USA). 

### 4.3. Real Time-qPCR

Total RNA was extracted from cell lines using Trizol (Sigma, St. Louis, MO, USA) as described previously [57]. The amount and purity of total RNA was determined using the Nanodrop 2000 spectrophotometer (Thermo Scientific, Wilmington, DE, USA). The A260/A280 ratio of RNA samples was calculated, and RNA samples with a ratio of ~1.8 were selected for cDNA synthesis. Of total RNA, 500nM was reverse transcribed using RevertAid First Strand cDNA Synthesis Kit (Thermo Fisher Scientific, Inc., Waltham, MA, USA) according to the manufacturer’s protocol. The cDNA samples were analyzed by qPCR for the expression of target genes using the following primers: NLRP3: Forward: 5’-ATGAGTGCTGCTTCGACATC-3’, Reverse: 5’-TTGTCACTCAGGTCCAGCTC-3’; Pro-CASP1: Forward: 5’-TGCCTTTCTTCTGGTCAGTG-3’, Reverse: 5’-TGCTGAGGTGAAGGAGAGAA-3’; and IL-1β: Forward: 5’-TCAGCACCTCTCAAGCAGAA-3’, Reverse: 5’-GGACTCTCTGGGTACAGCTC-3’. Measurements were normalized to the expression of a housekeeping gene, ACTB, using the following primers: Forward: 5’-GACAGGATGCAGAAGGAGATTACT-3’, Reverse: 5’-TGATCCACATCTGCTGGAAGGT-3’. The qPCR reaction mixture and the cycle parameters were described previously [58]. The threshold cycle (Ct) for each RNA expression was determined using the CFX384 Touch™ Real-Time PCR Detection System (Biorad, CA, USA). The 2-ΔCt method was used to calculate the fold change in gene expression.

### 4.4. Western Blot

Total protein was extracted using Sodium dodecyl sulfate (SDS) reducing buffer, separated on 8–12% gradient polyacrylamide gels, and transferred onto PVDF membranes (Biorad, CA, USA). Membranes were blocked with 5% non-fat milk for 30 min, followed by overnight incubation at 4 °C with a human anti-NLRP3 (1:300, Invitrogen, IL, USA). Membranes were washed with PBS containing 0.1% Tween 20 and incubated for 1 h at room temperature with the secondary antibody, anti-rabbit IgG (1:1000, Santa Cruz Biotechnology, Germany). Membranes were investigated using mouse anti-human Actin Beta-HRP conjugated antibody (1:1000, Sigma, St. Louis, MO, USA) as a control to normalize protein expression. Western blot results were visualized using Clarity Western ECL reagents and a ChemiDoc XRS + (Biorad, Hercules, CA, USA). Protein levels were quantified using ImageJ.

### 4.5. Enzyme-Linked Immunosorbent Assay (ELISA) 

The level of IL-1β in the cell-free supernatants was measured using a commercially available ELISA kit, according to manufacturer’s instructions (VECTOR-BEST, Novosibirsk, Russia), and a TECAN Infinite 200 Pro fluorimeter (Grödig, Austria). Each ELISA was completed in technical duplicates.

### 4.6. Real-Time Cell Proliferation Assay

PC3 and A549 cells (5 × 103) were seeded in each well of an E-plate 16 (ACEA Biosciences, San Diego, CA, USA), and the proliferation index of cells was monitored every 15 min for 96 h in real-time using the xCELLigence biosensor cell analysis system (ACEA Biosciences, San Diego, CA, USA).

### 4.7. Annexin V Assay

Cell viability was analysed using APC Annexin V Apoptosis Detection Kit with Propidium Iodide according to the manufacturer’s protocol (Sony Biotechnology, San Jose, CA, USA). Cells stained with Annexin V and PI were detected using a BD FACSAria III Flow Cytometer (BD Biosciences, San Diego, CA, USA) and the data processed with the FlowJo software package (FlowJo LLC, Ashland, OR, USA). Cells stained for Annexin-V only were considered as apoptotic. In contrast, those stained for both Annexin-V and PI were considered to be in late apoptosis phase or pyroptosis [59]. Cells that were negative for Annexin-V/PI were considered as alive. Experiments were performed three times per condition.

### 4.8. Cytokine Assay

The Bio-Plex Pro™ Human Chemokine Panel, 40-Plex, was used to analyze cell-free supernatants according to the manufacturer’s instructions. Fifty microliters of cell-free medium was collected from 24 h to 96 h of incubation and used to determine cytokine concentration. Data were analyzed using Luminex 200 analyzer with MasterPlex CT control software and MasterPlex QT analysis software (MiraiBio division of Hitachi Software San Francisco, CA, USA). Results are presented as a heatmap using the web-based program, Heatmapper (http://www.heatmapper.ca/ (accessed on 4 May 2021)) as described by Babicki and colleagues [60]. 

### 4.9. Statistical Analysis

Statistical analysis was performed using IBM SPSS Statistics for Windows, Version 20.0 (IBM Corp., Armonk, NY, USA). RT-qPCR, ELISA, Annexin V analyses were performed using the one-way ANOVA model with Tukey’s post hoc tests. Data are presented as mean ± SE. Statistical significance was set at a *p*-value < 0.05.

## 5. Conclusions

In conclusion, we, for the first time, analyzed the effect of the antibiotic doxycycline on the regulation of NLRP3 activation in cancer cells. We found that doxycycline inhibits LPS priming of NLRP3 and leads to early apoptosis, independent of the NLRP3 activating capacity of cancer cells. In vivo studies are required to validate the potential therapeutic benefit of using doxycycline to reduce inflammation within the tumor. We believe, that by reducing inflammation, it will have an additional beneficial effect on cancer therapy. Our findings highlight the potential of antibiotics as regulators of the NLRP3 inflammasome as a possible approach to avert cancer inflammation.

## Data Availability

Data is contained within the article and supplementary material.

## Supplementary Materials

The following are available online at https://www.mdpi.com/article/10.3390/ph14090852/s1, Figure S1: The effect of doxycycline on viability of non-tumor epithelial cells, Table S1: The effect of Doxycycline on mRNA expression of NLRP3 and downstream genes, Table S2: The effect of Doxycycline mediated NLRP3 suppression on the early phase of apoptosis, Table S3: The effect of Doxycycline on cytokine releasing pattern of PC3 cells, Table S4: The effect of Doxycycline on cytokine releasing pattern of A549 cells.
