# Supplementary material for: Doxycycline Attenuates Cancer Cell Growth by Suppressing NLRP3-Mediated Inflammation
Source: Pharmaceuticals (Basel). 2021 Aug 26;14(9):852. doi: 10.3390/ph14090852 (PMC8466018; doi:10.3390/ph14090852)
Supplement: Supplementary file 1 [file pharmaceuticals-14-00852-s001.zip › pharmaceuticals-1338584-supplementary.pdf]

Doxycycline Attenuates Cancer Cell Growth by Suppressing NLRP3 Mediated Inflammation

Supplementary Files:

Table S1: The effect of Doxycycline on mRNA expression of *NLRP3* and downstream genes.

mRNA	Comparisons		PC3					A549				
			Mean Difference	Std. Error	P*	95% CI Lower Bound	95% CI Upper Bound	Mean Difference	Std. Error	P*	95% CI Lower Bound	95% CI Upper Bound
NLRP3	U	G	-0.18	7.36	1.000	-24.89	24.53	-0.01	1.37	1.000	-4.62	4.60
		L	-15.50	7.36	0.345	-40.22	9.21	-7.18	1.37	**0.002**	-11.79	-2.57
		LN	-57.46	7.36	**<0.001**	-82.17	-32.74	-13.77	1.37	**<0.001**	-18.38	-9.16
		D	-31.33	7.36	**0.011**	-56.04	-6.62	-2.15	1.37	0.631	-6.76	2.45
		LD	-22.35	7.36	0.085	-47.07	2.36	0.04	1.37	1.000	-4.56	4.65
	G	L	-15.32	7.36	0.356	-40.04	9.39	-7.17	1.37	**0.002**	-11.78	-2.56
		LN	-57.28	7.36	**<0.001**	-81.99	-32.56	-13.76	1.37	**<0.001**	-18.37	-9.15
		D	-31.15	7.36	**0.011**	-55.86	-6.44	-2.15	1.37	0.634	-6.75	2.46
		LD	-22.17	7.36	0.089	-46.89	2.54	0.05	1.37	1.000	-4.56	4.66
	L	LN	-41.95	7.36	**0.001**	-66.01	-17.24	-6.59	1.37	**0.004**	-11.19	-1.98
		D	-15.83	7.36	0.326	-40.54	8.89	5.03	1.37	**0.030**	0.42	9.63
		LD	-6.85	7.36	0.931	-31.56	17.86	7.22	1.37	**0.002**	2.62	11.83
	LN	D	26.13	7.36	**0.036**	1.41	50.84	11.61	1.37	**<0.001**	7.01	16.22
		LD	35.10	7.36	**0.005**	10.39	59.82	13.81	1.37	**<0.001**	9.20	18.42
Pro-CASP1	U	G	-0.46	376.48	1.000	-131.06	121855.00	-0.33	2.55	1.000	-8.90	8.23
		L	-11.45	376.48	0.085	-240.96	11955.00	-10.75	2.55	**0.012**	-19.32	-2.18
		LN	-23.50	376.48	**<0.001**	-361.42	-108511.00	-12.65	2.55	**0.003**	-21.22	-4.08
		D	-23.53	376.48	**<0.001**	-361.76	-108845.00	-1.24	2.55	0.996	-9.81	7.33
		LD	-18.58	376.48	**0.004**	-312.26	-59345.00	-0.04	2.55	1.000	-8.61	8.53
	G	L	-10.99	376.48	0.103	-23.64	16.56	-10.41	2.55	**0.015**	-18.98	-1.85
		LN	-23.04	376.48	**0.001**	-356.82	-103911.00	-12.32	2.55	**0.004**	-20.88	-3.75
		D	-23.07	376.48	**0.001**	-35.72	-10.42	-0.90	2.55	0.999	-9.47	7.67
		LD	-18.12	376.48	**0.004**	-30.77	-5.47	0.30	2.55	1.000	-8.27	8.87
	L	LN	-12.05	376.48	0.065	-24.69	0.60	-1.90	2.55	0.972	-10.47	6.67
		D	-12.08	376.48	0.064	-24.73	0.57	9.51	2.55	**0.027**	0.94	18.08
		LD	-7.13	376.48	0.450	-19.78	55.16	10.71	2.55	**0.012**	2.14	19.28
	LN	D	-0.03	376.48	1.000	-12.68	126.12	11.41	2.55	**0.008**	2.84	19.98
		LD	4.92	376.48	0.777	-7.73	175.62	12.61	2.55	**0.004**	4.04	21.18
Pro-IL1β	U	G	0.05	1.17	1.000	-3.87	3.97	0.53	5.76	1.000	-18.80	19.86
		L	-7.20	1.17	**0.001**	-11.12	-3.28	-3.03	5.76	0.994	-22.36	16.31
		LN	-14.97	1.17	**<0.001**	-18.89	-11.05	-7.48	5.76	0.780	-26.81	11.86
		D	-1.30	1.17	0.867	-5.22	2.62	-9.38000-	5.76	0.596	-28.71	9.95
		LD	-2.64	1.17	0.280	-6.56	1.28	-44.92	5.76	**<0.001**	-64.25	-25.58
	G	L	-7.25	1.17	**<0.001**	-11.17	-3.33	-3.56	5.76	0.987	-22.89	15.78
		LN	-15.02	1.17	**<0.001**	-18.94	-11.10	-8.01	5.76	0.732	-27.34	11.33
		D	-1.35	1.17	0.850	-5.27	2.57	-9.91	5.76	0.544	-29.24	9.42
		LD	-2.69	1.17	0.265	-6.61	1.23	-45.45	5.76	**<0.001**	-64.78	-26.11
	L	LN	-7.77	1.17	**<0.001**	-11.69	-3.85	-4.45	5.76	0.967	-23.78	14.88
		D	5.90	1.17	**0.003**	1.98	9.82	-6.35	5.76	0.871	-25.69	12.98
		LD	4.56	1.17	**0.020**	0.64	8.48	-41.89	5.76	**<0.001**	-61.22	-22.56
	LN	D	13.67	1.17	**<0.001**	9.75	17.59	-1.90	5.76	0.999	-21.24	17.43
		LD	12.33	1.17	**<0.001**	8.41	16.25	-37.44	5.76	**<0.001**	-56.77	-18.11

*P-value calculated using One Way Anova and Tukey Test. P values less than 0.05 were considered significant and showed in bold. **U**: Untreated, **G**: Glibenclamide, **L**: LPS, **LN**: LPS-Nigericin, **D**: Doxycycline, **LD**: LPS-Doxycycline, n=3.

Table S2: The effect of Doxycycline mediated NLRP3 suppression on the early phase of apoptosis.

			Mean Difference	Std. Error	P*	95% CI Lower Bound	95% CI Upper Bound
PC3	U	L	-0.23	3.40	1.000	-11.13	10.66
		D	-43.97	3.40	**<0.001**	-54.86	-33.07
		LD	-74.33	3.40	**<0.001**	-85.23	-63.44
	L	D	-43.73	3.40	**<0.001**	-54.63	-32.84
		LD	-74.10	3.40	**<0.001**	-85.00	-63.20
	D	LD	-30.37	3.40	**<0.001**	-41.26	-19.47
A549	U	L	2.23	2.55	0.817	-5.92	10.39
		D	-43.97	2.55	**<0.001**	-52.12	-35.81
		LD	-49.60	2.55	**<0.001**	-57.76	-41.44
	L	D	-46.20	2.55	**<0.001**	-54.36	-38.04
		LD	-51.83	2.55	**<0.001**	-59.99	-43.68
	D	LD	-5.63	2.55	0.200	-13.79	2.52

*P-value calculated using One Way Anova and Tukey Test. P values less than 0.05 were considered significant and showed in bold. **U**: Untreated, **L**: LPS, **D**: Doxycycline, **LD**: LPS-Doxycycline, n=3.

Table S3: The effect of Doxycycline on cytokine releasing pattern of PC3 cells.

	24h						48h					
pg/mL	U	G	L	LN	D	LD	U	G	L	LN	D	LD
EGF	8.92	6.09	6.82	16.11	7.17	6.59	12.58	11.16	10.41	13.75	7.52	3.12
FGF-2	68.97	<1.46↓	49.43	788.49	<1.46↓	<1.46↓	87.07	41.36	78.03	152.86	70.86	49.43
Eotaxin	<2.27↓	<2.27↓	<2.27↓	<2.27↓	2.57	<2.27↓	<2.27↓	3.43	<2.27↓	<2.27↓	<2.27↓	<2.27↓
TGF-a	6.72	9.78	6.33	11.6	6.72	5.54	10.29	14.99	13.26	11.96	5.98	4.46
G-CSF	16534	16435	15394	3220	1865	7406	>18601 ↑	>18601 ↑	>18601 ↑	3576	3749	3130
Flt-3L	<2.49↓	<2.49↓	<2.49↓	<2.49↓	<2.49↓	<2.49↓	<2.49↓	<2.49↓	<2.49↓	<2.49↓	<2.49↓	<2.49↓
GM-CSF	3370	4352	3597	880.79	2712	2720	5072	6754	6198	920.85	3657	3072
Fractalkine	75.66	89.5	75.66	3.09	155.94	55.82	75.66	150.68	78.52	43.85	118.3	89.5
IFNa2	15.42	43.95	20.36	2.58	21.18	25.29	15.42	15.42	36.69	4.87	4.09	<1.71↓
IFN-y	<1.51↓	<1.51↓	<1.51↓	<1.51↓	<1.51↓	<1.51↓	<1.51↓	<1.51↓	<1.51↓	<1.51↓	<1.51↓	<1.51↓
GRO	>11613 ↑	>11613 ↑	>11613 ↑	>11613 ↑	>11613 ↑	>11613 ↑	>11613 ↑	>11613 ↑	>11613 ↑	>11613 ↑	>11613 ↑	>11613 ↑
IL-10	NAN	NAN	NAN	NAN	NAN	NAN	NAN	NAN	NAN	NAN	NAN	NAN
MCP-3	<1.82↓	<1.82↓	<1.82↓	<1.82↓	<1.82↓	<1.82↓	<1.82↓	<1.82↓	<1.82↓	<1.82↓	<1.82↓	<1.82↓
IL12p40	<1.77↓	<1.77↓	<1.77↓	<1.77↓	<1.77↓	<1.77↓	<1.77↓	<1.77↓	<1.77↓	<1.77↓	<1.77↓	<1.77↓
MDC	<0.82↓	<0.82↓	1.08	<0.82↓	<0.82↓	2.53	7.4	11.43	2.53	<0.82↓	<0.82↓	<0.82↓
IL12p70	NAN	NAN	NAN	NAN	NAN	NAN	NAN	NAN	NAN	NAN	NAN	NAN
PDGF-AA	16.57	26.6	16.09	<1.71↓	18.26	6.55	45.22	84.52	61.21	<1.71↓	15.08	21.83
IL-13	<1.56↓	<1.56↓	<1.56↓	<1.56↓	<1.56↓	<1.56↓	<1.56↓	<1.56↓	<1.56↓	<1.56↓	<1.56↓	<1.56↓
PDGF-AB/BB	<16.00 ↓	<16.00 ↓	<16.00 ↓	<16.00 ↓	<16.00 ↓	<16.00 ↓	<16.00 ↓	<16.00 ↓	<16.00 ↓	<16.00 ↓	<16.00 ↓	<16.00 ↓
IL-15	2.84	3.06	2.73	<1.30↓	3.86	2.84	8.35	10.74	10.61	<1.30↓	5.5	3.4
sCD40L	<1.92↓	<1.92↓	<1.92↓	<1.92↓	<1.92↓	<1.92↓	<1.92↓	2.85	<1.92↓	<1.92↓	<1.92↓	<1.92↓
IL-17A	<1.70↓	<1.70↓	<1.70↓	<1.70↓	<1.70↓	<1.70↓	<1.70↓	<1.70↓	<1.70↓	<1.70↓	<1.70↓	<1.70↓
IL1Ra	<3.98↓	<3.98↓	<3.98↓	<3.98↓	<3.98↓	<3.98↓	<3.98↓	<3.98↓	<3.98↓	<3.98↓	<3.98↓	<3.98↓
IL1a	14.25	17.16	23.05	372.39	29.67	8.59	61.44	25.26	64.13	435.38	78.61	28.94
IL-9	15.34	8.98	9.8	42.33	13.98	6.36	20.3	17.35	18.76	34.26	11.15	5.67
IL-1b	<1.40↓	<1.40↓	<1.40↓	9.28	1.83	<1.40↓	5.43	3.76	5.88	2.05	4.67	3.39
IL-2	<1.57↓	<1.57↓	<1.57↓	<1.57↓	<1.57↓	<1.57↓	<1.57↓	<1.57↓	<1.57↓	<1.57↓	<1.57↓	<1.57↓
IL-3	<1.53↓	<1.53↓	<1.53↓	<1.53↓	<1.53↓	<1.53↓	<1.53↓	<1.53↓	<1.53↓	<1.53↓	<1.53↓	<1.53↓
IL-4	20.21	16.07	17.1	12.69	18.13	17.44	21.61	20.21	20.21	13.36	14.04	11.36
IL-5	<2.00↓	<2.00↓	<2.00↓	<2.00↓	<2.00↓	<2.00↓	<2.00↓	<2.00↓	<2.00↓	<2.00↓	<2.00↓	<2.00↓
IL-6	10.07	16.08	8.72	6.01	18.87	6.67	18.72	41.73	21.34	6.6	32.6	32
IL-7	<1.84↓	<1.84↓	<1.84↓	<1.84↓	<1.84↓	<1.84↓	<1.84↓	<1.84↓	<1.84↓	<1.84↓	<1.84↓	<1.84↓
IL-8	>7775↑	>7775↑	>7775↑	6395	>7775↑	>7775↑	>7775↑	>7775↑	>7775↑	7634	>7775↑	>7775↑
IP10	49.87	21.93	22.19	20.88	49.78	26.71	103.47	26.46	80.98	12.99	24.73	18.46
MCP-1	801.21	406.41	736.66	120.1	329.1	258.37	2042	639.97	1799	129.41	168.14	204.74
MIP-1a	<2.12↓	<2.12↓	<2.12↓	<2.12↓	<2.12↓	<2.12↓	<2.12↓	2.29	<2.12↓	<2.12↓	<2.12↓	<2.12↓
MIP-1b	<1.79↓	<1.79↓	<1.79↓	4.17	<1.79↓	<1.79↓	<1.79↓	2.98	<1.79↓	<1.79↓	<1.79↓	<1.79↓
Rantes	<6.13↓	<6.13↓	<6.13↓	<6.13↓	<6.13↓	<6.13↓	<6.13↓	<6.13↓	<6.13↓	<6.13↓	<6.13↓	<6.13↓
TNFa	41.43	51.09	40.38	32.74	24.49	13.46	115.28	148.35	122.09	19.46	68.68	64.97
TNF-b	<1.93↓	<1.93↓	<1.93↓	<1.93↓	<1.93↓	<1.93↓	<1.93↓	<1.93↓	<1.93↓	<1.93↓	<1.93↓	<1.93↓
VEGF	63.34	169.58	60.19	23.72	73.68	54.82	195.92	460.01	194.04	23.72	160.3	132.8

	72h						96 h					
	U	G	L	LN	D	LD	U	G	L	LN	D	LD
EGF	10.22	14.08	8.5	10.5	6.09	3.91	10.88	9.63	13.92	6.09	2.61	9.43
FGF-2	214.37	83.88	305.16	35.21	303.28	230.27	1156	246.54	658.74	<1.46↓	389.07	266.91
Eotaxin	2.33	2.57	<2.27↓	<2.27↓	<2.27↓	<2.27↓	<2.27↓	<2.27↓	<2.27↓	<2.27↓	<2.27↓	<2.27↓
TGF-a	10.03	25.54	8.46	10.18	11.19	7.08	14.61	23.33	10.09	9.85	8.62	8.25
G-CSF	>18601 ↑	>18601 ↑	>18601 ↑	2312	6741	2261	>18601 ↑	15627	>18601 ↑	2475	784.9	5606
Flt-3L	<2.49↓	<2.49↓	<2.49↓	<2.49↓	<2.49↓	<2.49↓	<2.49↓	<2.49↓	<2.49↓	<2.49↓	<2.49↓	<2.49↓
GM-CSF	4885	8890	4193	738.56	4836	3678	7420	7204	5751	697.65	2790	4000
Fractalkine	43.85	54.18	43.85	40.19	57.44	40.19	43.85	34.46	24	<2.01↓	34.46	68.33
IFNa2	20.36	39.93	12.95	2.58	7.25	5.65	36.69	17.07	23.64	7.25	<1.71↓	<1.71↓
IFN-y	<1.51↓	<1.51↓	<1.51↓	<1.51↓	<1.51↓	<1.51↓	<1.51↓	<1.51↓	<1.51↓	<1.51↓	<1.51↓	<1.51↓
GRO	>11613 ↑	>11613 ↑	>11613 ↑	>11613 ↑	>11613 ↑	>11613 ↑	>11613 ↑	>11613 ↑	>11613 ↑	>11613 ↑	>11613 ↑	>11613 ↑
IL-10	NAN	NAN	NAN	NAN	NAN	NAN	NAN	NAN	NAN	NAN	NAN	NAN
MCP-3	<1.82↓	<1.82↓	<1.82↓	<1.82↓	<1.82↓	<1.82↓	<1.82↓	<1.82↓	<1.82↓	<1.82↓	<1.82↓	<1.82↓
IL12p40	<1.77↓	<1.77↓	<1.77↓	<1.77↓	<1.77↓	<1.77↓	<1.77↓	<1.77↓	<1.77↓	<1.77↓	<1.77↓	<1.77↓
MDC	2.53	4.5	5.11	<0.82↓	<0.82↓	<0.82↓	8.46	<0.82↓	1.08	<0.82↓	<0.82↓	<0.82↓
IL12p70	NAN	NAN	NAN	NAN	NAN	NAN	NAN	NAN	NAN	NAN	NAN	NAN
PDGF-AA	37.27	169.56	37.21	<1.71↓	27.97	5.71	80.88	143.11	73.97	<1.71↓	31.31	30.73

IL-13	<1.56↓	<1.56↓	<1.56↓	<1.56↓	<1.56↓	<1.56↓	<1.56↓	<1.56↓	<1.56↓	<1.56↓	<1.56↓	<1.56↓
PDGF-AB/BB	<16.00 ↓	<16.00 ↓	<16.00 ↓	<16.00 ↓	<16.00 ↓	<16.00 ↓	<16.00 ↓	<16.00 ↓	<16.00 ↓	<16.00 ↓	<16.00 ↓	<16.00 ↓
IL-15	7.8	20.76	7.12	<1.30↓	10.42	7.19	16.35	17.22	13.58	<1.30↓	5.14	7.19
sCD40L	<1.92↓	<1.92↓	<1.92↓	<1.92↓	<1.92↓	<1.92↓	<1.92↓	<1.92↓	<1.92↓	<1.92↓	<1.92↓	<1.92↓
IL-17A	<1.70↓	<1.70↓	<1.70↓	<1.70↓	<1.70↓	<1.70↓	<1.70↓	<1.70↓	<1.70↓	<1.70↓	<1.70↓	<1.70↓
IL1Ra	<3.98↓	<3.98↓	<3.98↓	<3.98↓	<3.98↓	<3.98↓	<3.98↓	<3.98↓	<3.98↓	<3.98↓	<3.98↓	<3.98↓
IL1a	131.61	187.7	499.46	258.97	388.97	415.29	848.72	279.57	500.54	312.46	589.55	447.71
IL-9	14.45	13.57	15.87	36.6	11.59	6.79	21.19	10.68	19.89	13.01	10.27	11.83
IL-1b	6.95	12.85	15.01	<1.40↓	26.7	20.55	95.38	41.36	30.6	<1.40↓	42.13	21.64
IL-2	<1.57↓	<1.57↓	<1.57↓	<1.57↓	<1.57↓	<1.57↓	<1.57↓	<1.57↓	<1.57↓	<1.57↓	<1.57↓	<1.57↓
IL-3	<1.53↓	<1.53↓	<1.53↓	<1.53↓	<1.53↓	<1.53↓	<1.53↓	<1.53↓	<1.53↓	<1.53↓	<1.53↓	<1.53↓
IL-4	20.91	23.01	18.13	11.03	14.71	15.39	20.91	13.36	16.07	10.71	10.05	10.71
IL-5	<2.00↓	<2.00↓	<2.00↓	<2.00↓	<2.00↓	<2.00↓	<2.00↓	<2.00↓	<2.00↓	<2.00↓	<2.00↓	<2.00↓
IL-6	22.92	108.22	40.66	<1.82↓	110.21	62.3	174.78	125.04	101.87	<1.82↓	123.65	57.01
IL-7	<1.84↓	2.45	<1.84↓	<1.84↓	2.2	<1.84↓	<1.84↓	<1.84↓	<1.84↓	<1.84↓	<1.84↓	<1.84↓
IL-8	>7775↑	>7775↑	>7775↑	5772	>7775↑	>7775↑	>7775↑	>7775↑	>7775↑	5794	>7775↑	>7775↑
IP10	56.93	55.81	189.54	13.59	56.84	22.7	353.53	55.15	293.85	13.59	50.36	70.22
MCP-1	925.58	767.53	1473	81.47	656.81	126.04	3454	792.33	2138	88.95	316.61	701.96
MIP-1a	<2.12↓	<2.12↓	<2.12↓	<2.12↓	<2.12↓	<2.12↓	<2.12↓	<2.12↓	<2.12↓	<2.12↓	<2.12↓	<2.12↓
MIP-1b	<1.79↓	5.29	<1.79↓	<1.79↓	<1.79↓	<1.79↓	8.39	<1.79↓	<1.79↓	<1.79↓	<1.79↓	<1.79↓
Rantes	<6.13↓	<6.13↓	<6.13↓	<6.13↓	<6.13↓	<6.13↓	<6.13↓	<6.13↓	<6.13↓	<6.13↓	<6.13↓	<6.13↓
TNFa	120.47	262.66	208.17	16.53	117.06	76.92	337.13	340.85	348.8	9.73	58.84	67.39
TNF-b	<1.93↓	<1.93↓	<1.93↓	<1.93↓	<1.93↓	<1.93↓	<1.93↓	<1.93↓	<1.93↓	<1.93↓	<1.93↓	<1.93↓
VEGF	173.83	917.85	338.19	3.3	331.52	163.4	418.91	613.82	439.61	6.27	132.32	217.48

Table S4: The effect of Doxycycline on cytokine releasing pattern of A549 cells.

	24h						48h					
	U	G	L	LN	D	LD	U	G	L	LN	D	LD
EGF	4.78	2.25	3.6	<2.22↓	2.61	<2.22↓	4.78	13.75	3.28	3.44	<2.22↓	<2.22↓
FGF-2	144.76	86.28	221.28	3527	7.91	<1.46↓	326.95	311.38	264.88	848.85	93.91	124.48
Eotaxin	<2.27↓	<2.27↓	<2.27↓	<2.27↓	<2.27↓	<2.27↓	<2.27↓	~~N/A~~	<2.27↓	<2.27↓	<2.27↓	<2.27↓
TGF-a	<1.63↓	<1.63↓	<1.63↓	3.49	<1.63↓	<1.63↓	<1.63↓	<1.63↓	<1.63↓	3.62	<1.63↓	<1.63↓
G-CSF	143.8	169.2	173.47	49.3	42.03	38.05	390.74	241.93	366.93	97.09	65.54	54.8
Flt-3L	<2.49↓	<2.49↓	<2.49↓	<2.49↓	<2.49↓	<2.49↓	<2.49↓	8.76	<2.49↓	<2.49↓	<2.49↓	<2.49↓
GM-CSF	42.82	46.79	41.5	15.49	21.73	14.31	62.14	35.84	61	16.68	32.84	24.83
Fractalkine	97.37	47.39	50.83	36.41	11.76	<2.01↓	50.83	3.09	57.44	21.73	32.46	54.18
IFNa2	14.6	15.42	18.71	18.71	18.71	22	17.89	~~N/A~~	25.29	10.5	15.42	8.87
IFN-γ	<1.51↓	<1.51↓	<1.51↓	<1.51↓	<1.51↓	<1.51↓	<1.51↓	<1.51↓	<1.51↓	<1.51↓	<1.51↓	<1.51↓
GRO	>11613 ↑	>11613 ↑	>11613 ↑	1161	603.09	626.43	>11613 ↑	10944	>11613 ↑	1028	1856	1963
IL-10	NAN	NAN	NAN	NAN	NAN	NAN	NAN	~~N/A~~	NAN	NAN	NAN	NAN
MCP-3	<1.82↓	<1.82↓	<1.82↓	<1.82↓	<1.82↓	<1.82↓	<1.82↓	<1.82↓	<1.82↓	<1.82↓	<1.82↓	<1.82↓
IL12p40	<1.77↓	<1.77↓	<1.77↓	<1.77↓	<1.77↓	<1.77↓	<1.77↓	<1.77↓	<1.77↓	<1.77↓	<1.77↓	<1.77↓
MDC	<0.82↓	<0.82↓	3.87	<0.82↓	<0.82↓	<0.82↓	3.87	8.46	7.4	<0.82↓	<0.82↓	<0.82↓
IL12p70	NAN	NAN	NAN	NAN	NAN	NAN	NAN	~~N/A~~	NAN	NAN	NAN	NAN
PDGF-AA	591.57	541.38	684.61	115.97	226.27	219.62	1651	487.4	1200	153.51	453.86	450.62
IL-13	<1.56↓	<1.56↓	<1.56↓	<1.56↓	<1.56↓	<1.56↓	<1.56↓	17.77	<1.56↓	<1.56↓	<1.56↓	<1.56↓
PDGF-AB/BB	<16.00 ↓	<16.00 ↓	<16.00 ↓	<16.00 ↓	<16.00 ↓	<16.00 ↓	<16.00 ↓	<16.00 ↓	<16.00 ↓	<16.00 ↓	<16.00 ↓	<16.00 ↓
IL-15	2.06	2.95	2.73	<1.30↓	2.5	1.31	8.17	3.97	5.26	<1.30↓	3.97	2.95
sCD40L	<1.92↓	<1.92↓	<1.92↓	<1.92↓	<1.92↓	<1.92↓	<1.92↓	<1.92↓	<1.92↓	<1.92↓	<1.92↓	<1.92↓
IL-17A	<1.70↓	<1.70↓	<1.70↓	<1.70↓	<1.70↓	<1.70↓	<1.70↓	<1.70↓	<1.70↓	<1.70↓	<1.70↓	<1.70↓
IL1Ra	<3.98↓	<3.98↓	<3.98↓	<3.98↓	<3.98↓	<3.98↓	<3.98↓	~~N/A~~	<3.98↓	<3.98↓	<3.98↓	<3.98↓
IL1a	<1.55↓	<1.55↓	8.59	<1.55↓	<1.55↓	<1.55↓	<1.55↓	<1.55↓	<1.55↓	<1.55↓	<1.55↓	<1.55↓
IL-9	7.17	2.31	5.84	<1.67↓	3.44	2.6	9.36	<1.67↓	5.73	7.4	4.92	4.47
IL-1b	<1.40↓	<1.40↓	<1.40↓	<1.40↓	<1.40↓	<1.40↓	<1.40↓	1106	<1.40↓	<1.40↓	<1.40↓	<1.40↓
IL-2	<1.57↓	<1.57↓	<1.57↓	<1.57↓	<1.57↓	<1.57↓	<1.57↓	<1.57↓	<1.57↓	<1.57↓	<1.57↓	<1.57↓
IL-3	<1.53↓	<1.53↓	<1.53↓	<1.53↓	<1.53↓	<1.53↓	<1.53↓	~~N/A~~	<1.53↓	<1.53↓	<1.53↓	<1.53↓
IL-4	10.71	12.69	15.05	<2.03↓	<2.03↓	<2.03↓	15.73	12.36	14.37	<2.03↓	<2.03↓	<2.03↓
IL-5	<2.00↓	<2.00↓	<2.00↓	<2.00↓	<2.00↓	<2.00↓	<2.00↓	<2.00↓	<2.00↓	<2.00↓	<2.00↓	<2.00↓
IL-6	27.41	25.19	32.09	<1.82↓	9.3	6.1	104.25	40.42	89.86	<1.82↓	42.64	35.95
IL-7	<1.84↓	<1.84↓	<1.84↓	<1.84↓	<1.84↓	<1.84↓	<1.84↓	4.78	<1.84↓	<1.84↓	<1.84↓	<1.84↓
IL-8	2437	2771	3135	261.27	669.31	513.44	7144	3677	6551	235.02	2017	2033
IP10	21.41	18.18	26.46	4.43	5.5	5.32	53.73	~~N/A~~	38.76	2.27	9.55	6.19

MCP-1	5687	>7400↑	5682	1629	1158	1258	5678	3286	6344	2439	3874	2631
MIP-1a	<2.12↓	<2.12↓	<2.12↓	<2.12↓	<2.12↓	<2.12↓	<2.12↓	4.5	<2.12↓	<2.12↓	<2.12↓	<2.12↓
MIP-1b	<1.79↓	<1.79↓	<1.79↓	<1.79↓	<1.79↓	<1.79↓	<1.79↓	<1.79↓	<1.79↓	<1.79↓	<1.79↓	<1.79↓
Rantes	<6.13↓	<6.13↓	<6.13↓	<6.13↓	<6.13↓	<6.13↓	<6.13↓	<6.13↓	<6.13↓	<6.13↓	<6.13↓	<6.13↓
TNFa	<1.65↓	<1.65↓	1.78	<1.65↓	<1.65↓	<1.65↓	2.42	<1.65↓	2.75	<1.65↓	<1.65↓	<1.65↓
TNF-b	<1.93↓	<1.93↓	<1.93↓	<1.93↓	<1.93↓	<1.93↓	<1.93↓	<1.93↓	<1.93↓	<1.93↓	<1.93↓	<1.93↓
VEGF	430.16	781.35	554.29	94.61	351.07	271.05	1413	800.04	957.66	115.92	922.33	780.1
	72h						96 h					
	U	G	L	LN	D	LD	U	G	L	LN	D	LD
EGF	3.28	2.61	4.87	3.92	<2.22↓	3.14	6.7	5.52	5.09	4.64	3.14	3.14
FGF-2	912.59	905.47	1350	167.29	626.92	470.52	1975	2061	1915	63.64	732.97	1227
Eotaxin	<2.27↓	<2.27↓	3.18	<2.22↓	<2.27↓	<2.22↓	<2.22↓	2.94	<2.22↓	<2.22↓	<2.22↓	<2.22↓
TGF-a	2.09	3.47	<1.89↓	4.27	<1.63↓	<1.89↓	<1.89↓	5.32	<1.89↓	5.89	<1.89↓	2.13
G-CSF	295.3	618.91	757.02	92.02	60.21	96.35	1264	1329	1128	107.49	95.28	111.13
Flt-3L	<2.49↓	<2.49↓	<1.76↓	<1.76↓	<2.49↓	<1.76↓	<1.76↓	<1.76↓	<1.76↓	<1.76↓	<1.76↓	<1.76↓
GM-CSF	78.87	76.75	92.9	29.27	33.26	66.39	127.31	156.66	131.26	28.16	59.62	101.36
Fractalkine	32.46	19.39	28.13	6.3	19.39	25.05	59.4	59.4	37.14	37.14	31.17	40.06
IFNa2	26.92	16.24	32.91	9.64	12.13	20.38	58.28	34.67	31.14	34.67	27.58	31.14
IFN-y	<1.51↓	<1.51↓	2.12	<1.66↓	<1.51↓	<1.66↓	<1.66↓	2.12	<1.66↓	<1.66↓	<1.66↓	<1.66↓
GRO	>11613↑	>11613↑	>14776↑	1134	2364	6987	>14776↑	>14776↑	>14776↑	1262	9187	>14776↑
IL-10	NAN	NAN	<1.06↓	<1.06↓	NAN	<1.06↓	<1.06↓	<1.06↓	<1.06↓	<1.06↓	<1.06↓	<1.06↓
MCP-3	<1.82↓	<1.82↓	<1.86↓	<1.86↓	<1.82↓	<1.86↓	<1.86↓	<1.86↓	<1.86↓	<1.86↓	<1.86↓	<1.86↓
IL12p40	<1.77↓	<1.77↓	<2.14↓	<2.14↓	<1.77↓	<2.14↓	<2.14↓	<2.14↓	<2.14↓	<2.14↓	<2.14↓	<2.14↓
MDC	5.11	<0.82↓	7.42	<1.64↓	<0.82↓	<1.64↓	9.33	6.42	11.6	<1.64↓	<1.64↓	<1.64↓
IL12p70	NAN	NAN	<1.48↓	<1.48↓	NAN	<1.48↓	<1.48↓	<1.48↓	<1.48↓	<1.48↓	<1.48↓	<1.48↓
PDGF-AA	994.91	1112	2134	198.69	330.57	1038	4876	2432	4024	220.65	594.61	1422
IL-13	<1.56↓	<1.56↓	<1.20↓	<1.20↓	<1.56↓	<1.20↓	<1.20↓	<1.20↓	<1.20↓	<1.20↓	<1.20↓	<1.20↓
PDGF-AB/BB	<16.00↓	<16.00↓	<1.66↓	<1.66↓	<16.00↓	<1.66↓	<1.66↓	<1.66↓	<1.66↓	<1.66↓	<1.66↓	167.07
IL-15	4.79	6.7	14.29	<1.73↓	3.74	12.88	25.34	25.98	23.98	<1.73↓	13.23	17.4
sCD40L	<1.92↓	<1.92↓	1.88	<1.67↓	<1.92↓	<1.67↓	<1.67↓	1.88	<1.67↓	<1.67↓	<1.67↓	<1.67↓
IL-17A	<1.70↓	<1.70↓	<2.02↓	<2.02↓	<1.70↓	<2.02↓	<2.02↓	<2.02↓	<2.02↓	<2.02↓	<2.02↓	<2.02↓
IL1Ra	<3.98↓	<3.98↓	<1.72↓	<1.72↓	<3.98↓	<1.72↓	<1.72↓	<1.72↓	<1.72↓	<1.72↓	<1.72↓	<1.72↓
IL1a	3.51	3.51	<1.62↓	<1.62↓	<1.55↓	<1.62↓	47.04	90.19	30.75	17.08	3.47	22.26
IL-9	8.63	4.27	4.56	7.66	4.02	<2.11↓	10.71	3.27	3.98	10.3	4.19	2.51
IL-1b	<1.40↓	<1.40↓	<1.80↓	<1.80↓	<1.40↓	<1.80↓	<1.80↓	<1.80↓	<1.80↓	<1.80↓	<1.80↓	<1.80↓
IL-2	<1.57↓	<1.57↓	<2.01↓	<2.01↓	<1.57↓	<2.01↓	<2.01↓	<2.01↓	<2.01↓	<2.01↓	<2.01↓	<2.01↓
IL-3	<1.53↓	<1.53↓	<1.88↓	<1.88↓	<1.53↓	<1.88↓	<1.88↓	<1.88↓	<1.88↓	<1.88↓	<1.88↓	<1.88↓
IL-4	14.04	15.39	31.72	<2.06↓	<2.03↓	8.57	32.91	46.08	38.3	<2.06↓	9.65	24.58
IL-5	<2.00↓	<2.00↓	<1.61↓	<1.61↓	<2.00↓	<1.61↓	<1.61↓	<1.61↓	<1.61↓	<1.61↓	<1.61↓	<1.61↓
IL-6	98.56	77.74	192.39	2.78	67.03	102.42	371.73	221	340.4	<1.94↓	142.77	223.62
IL-7	<1.84↓	<1.84↓	<2.19↓	<2.19↓	<1.84↓	<2.19↓	<2.19↓	3.94	<2.19↓	<2.19↓	<2.19↓	<2.19↓
IL-8	6674	6061	>5739↑	321.68	3539	>5739↑	>5739↑	>5739↑	>5739↑	368.51	>5739↑	>5739↑
IP10	21.41	20.62	56.98	3.37	10.51	12.83	63.93	30.74	82.64	5.53	13.38	15.91
MCP-1	6491	7107	>7382↑	2230	2166	>7382↑	6505	>7382↑	>7382↑	3464	6823	>7382↑
MIP-1a	<2.12↓	<2.12↓	<1.96↓	<1.96↓	<2.12↓	<1.96↓	2.26	2.7	<1.96↓	<1.96↓	<1.96↓	<1.96↓
MIP-1b	<1.79↓	<1.79↓	<1.72↓	<1.72↓	<1.79↓	<1.72↓	3.32	6.92	5.18	<1.72↓	<1.72↓	<1.72↓
Rantes	26.23	<6.13↓	<2.71↓	<2.71↓	<6.13↓	<2.71↓	139.77	<2.71↓	153.11	<2.71↓	<2.71↓	<2.71↓
TNFa	2.69	<1.65↓	3.89	<1.61↓	<1.65↓	<1.61↓	4.42	4.77	4.6	<1.61↓	<1.61↓	<1.61↓
TNF-b	<1.93↓	<1.93↓	<1.69↓	<1.69↓	<1.93↓	<1.69↓	<1.69↓	<1.69↓	<1.69↓	<1.69↓	<1.69↓	<1.69↓
VEGF	1503	4932	3272	137.71	751.98	2710	>6506↑	>6506↑	>6506↑	135.94	2394	6305

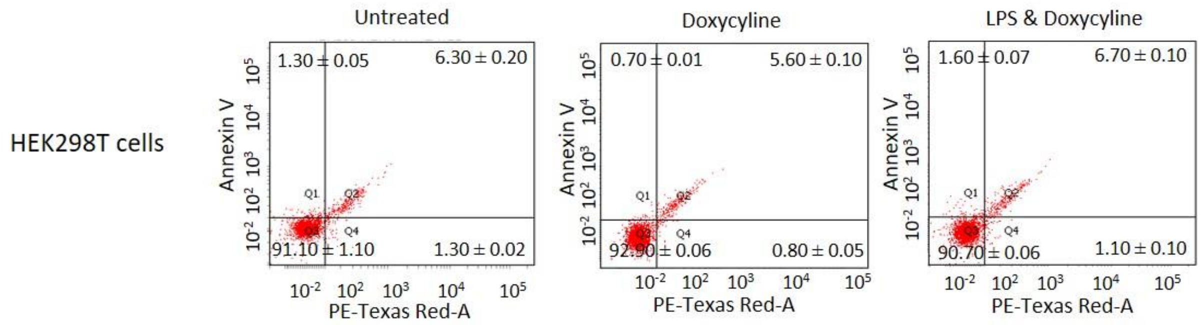

Figure S1: The effect of doxycycline on viability of non-tumor epithelial cells.

The original images of western blots

A549-marker

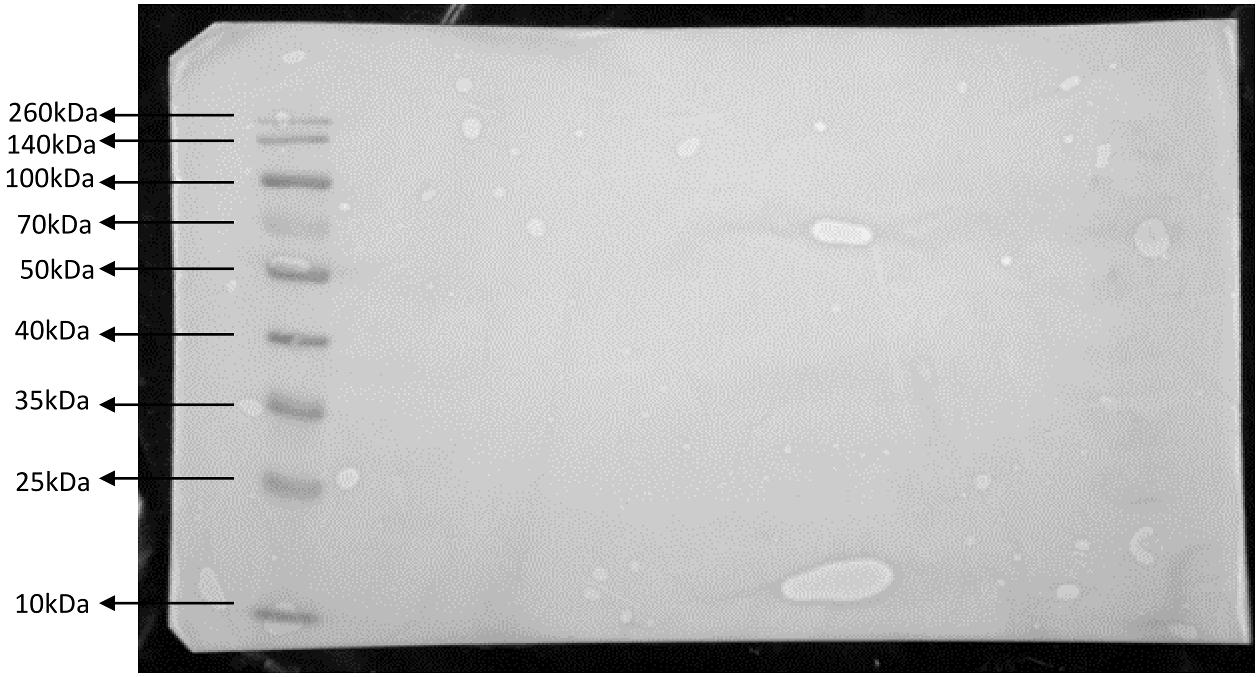

A549-Actb

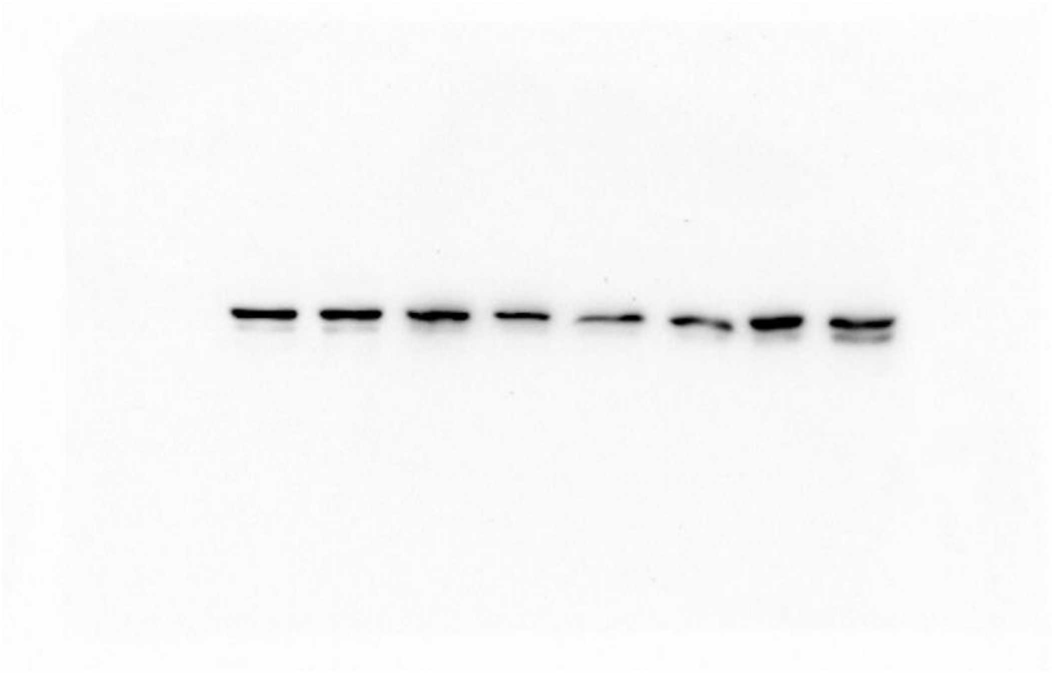

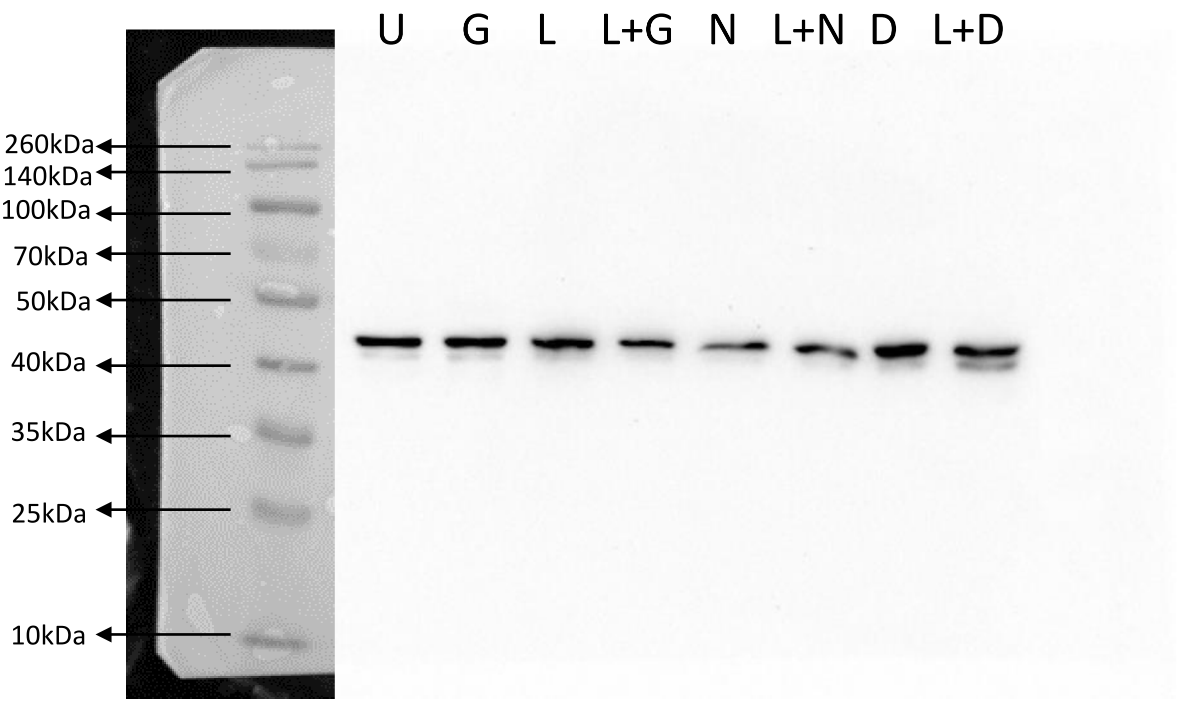
U
G
L
L+G
N
L+N
D
L+D
260kDa
140kDa
100kDa
70kDa
50kDa
40kDa
35kDa
25kDa
10kDa

A549 NLRP3

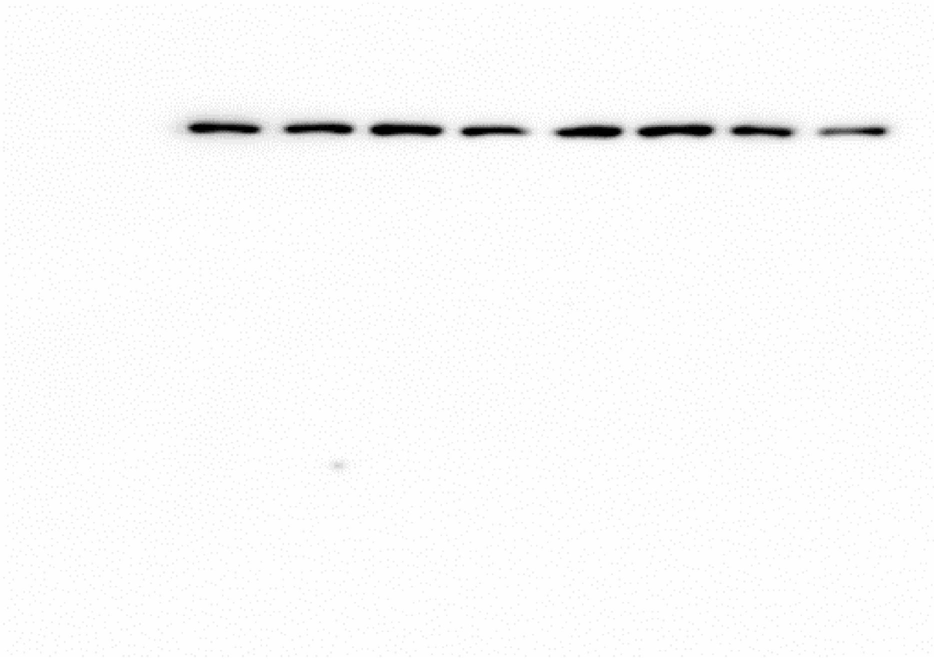

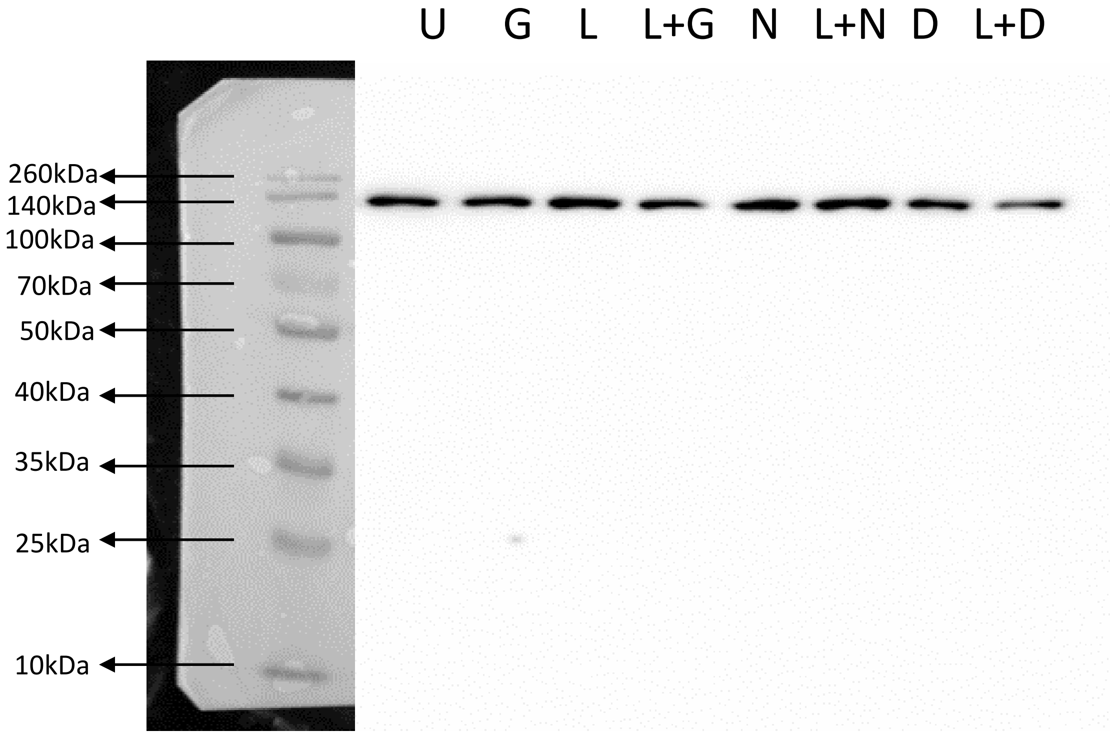
U
G
L
L+G
N
L+N
D
L+D
260kDa
140kDa
100kDa
70kDa
50kDa
40kDa
35kDa
25kDa
10kDa

PC3 marker

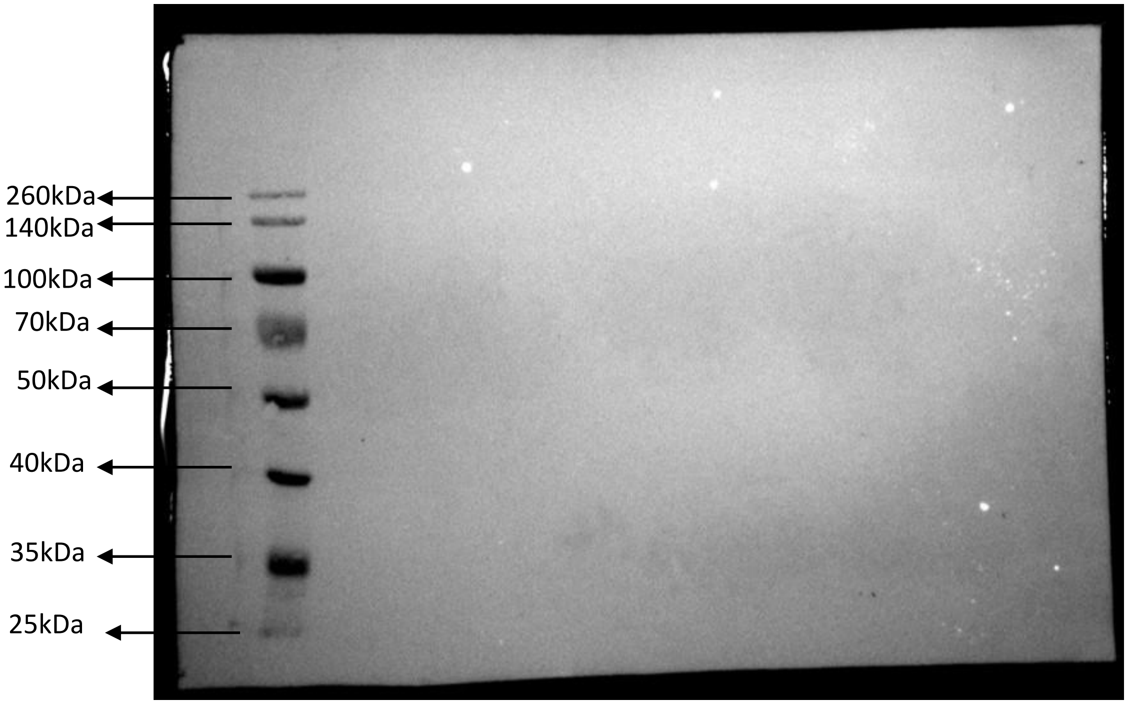

PC3 Actb

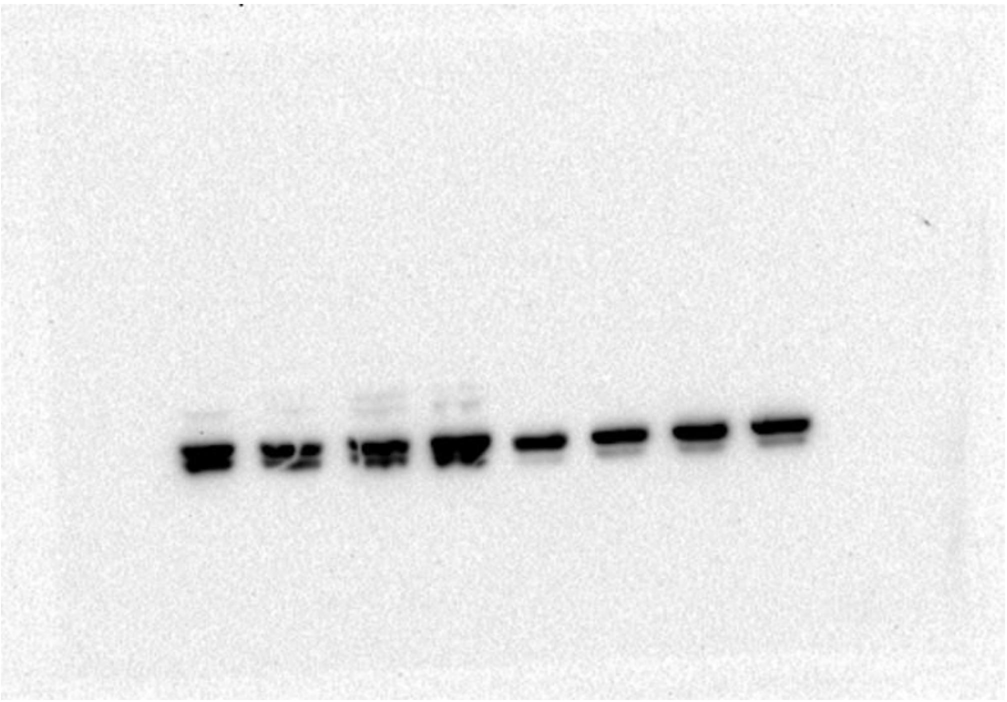

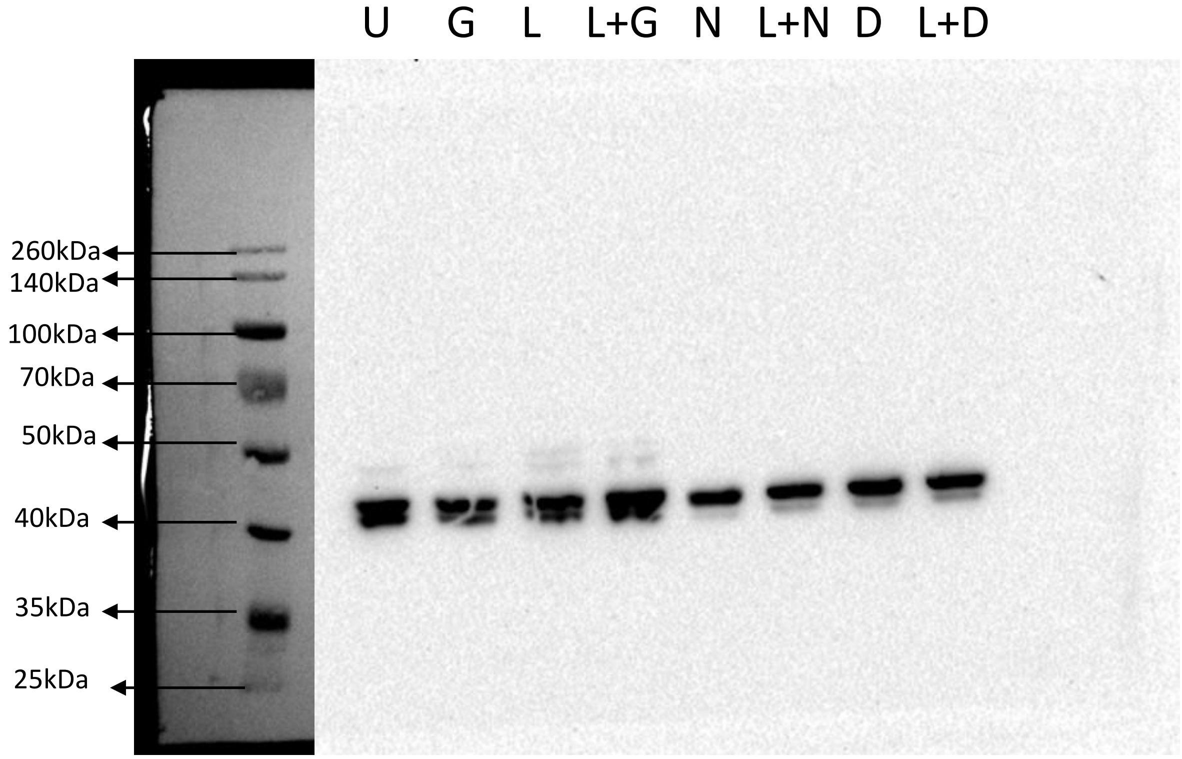
U
G
L
L+G
N
L+N
D
L+D
260kDa
140kDa
100kDa
70kDa
50kDa
40kDa
35kDa
25kDa

PC3 NLRP3

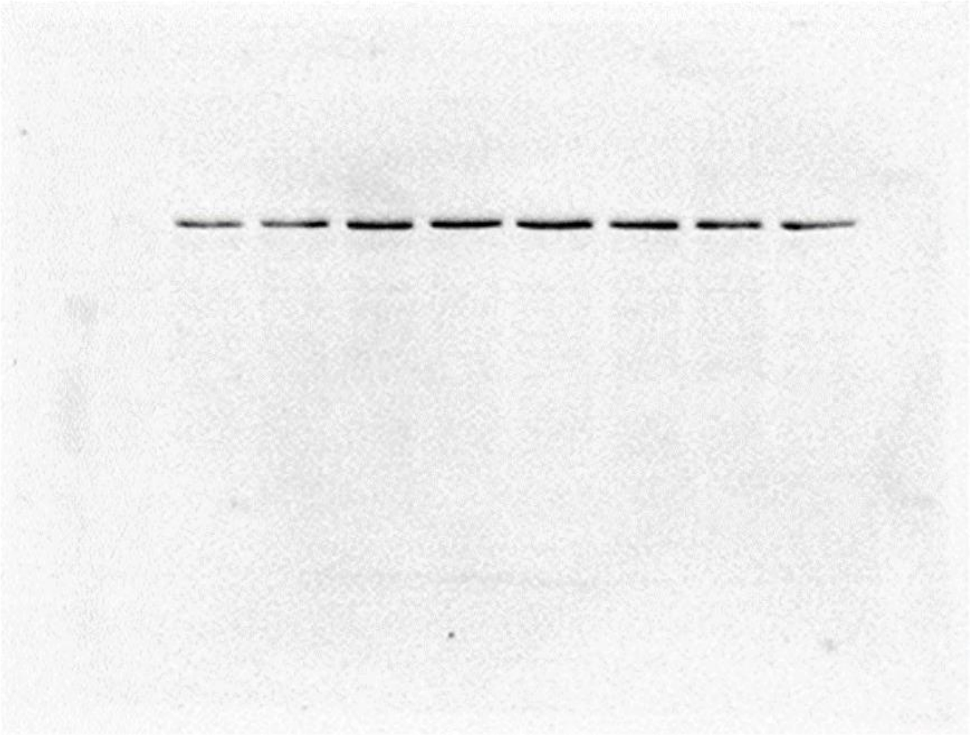

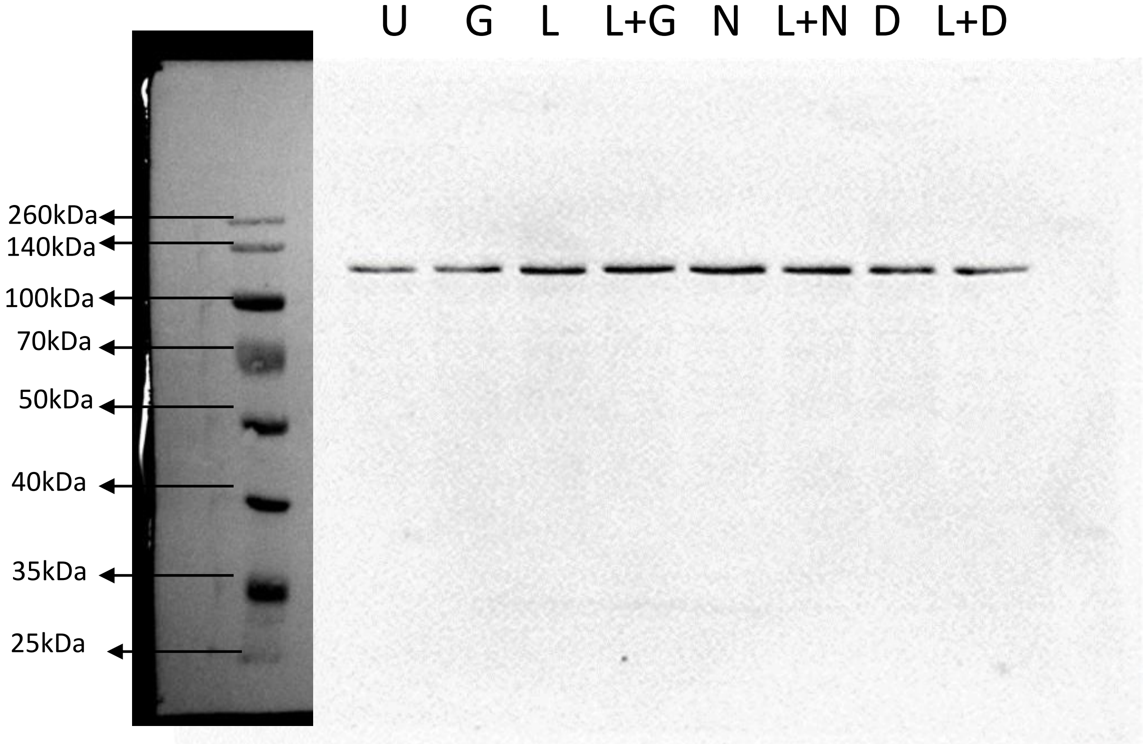
U
G
L
L+G
N
L+N
D
L+D
260kDa
140kDa
100kDa
70kDa
50kDa
40kDa
35kDa
25kDa
